# Towards cultural validation of the revised children’s anxiety and depression scale in Karnataka, India: Think aloud interviews among 13–17 year olds

**DOI:** 10.1371/journal.pone.0352705

**Published:** 2026-08-19

**Authors:** Amy Palmer, Daryl B. O’Connor, Poornima Bhola, Prachi Khandeparkar, N. Janardhana, Sphoorthi Prabhu, Krupa A. L, Mutharaju Arelingaiah, Jayalaxmi K. P, Ritwika Nag, Siobhan Hugh-Jones

**Affiliations:** 1 School of Psychology, University of Leeds, Leeds, England; 2 NIMHANS, Bangalore, Karnataka, India; 3 Sangath India, Psychology, Goa, India; Taipei Veterans General Hospital, TAIWAN

## Abstract

Improving youth mental health is a national priority in India, especially given the very high rates of youth suicide. Yet prevalence data in India is often incomplete and more culturally validated mental health measures are needed to inform prevention and intervention work. The Revised Children’s Anxiety and Depression Scale (RCADS-47) is a widely-used measure of mental health globally, including in India, but is yet to be culturally validated there. As the first step in establishing cultural validity, this study aimed to establish the cultural and semantic equivalence of the English-version of the RCADS-47 in a sample of Indian adolescents. This study examined Indian adolescents’ (*n* = 12) understanding of the measure items and response meaning via Think Aloud interviews. Responses to each item were coded according to predefined categories representing comprehension. Items coded as problematic for three or more participants were considered for rephrasing. Of the 47 items, 14 were identified as problematic, with the Obsessive-Compulsive Disorder subscale presenting the most problems. Several RCADS-47 items were identified as potentially problematic for Indian adolescents. Potential causes of these difficulties and suggested rephrasing are discussed along with the next stage of validating the measure in India.

## 1. Introduction

Adolescent mental health is of concern in India. An estimated 9.8 million 13–17-year olds in India have a mental health disorder [1], of which anxiety and depression are the most common [2]. India also has one of the highest youth suicide rates in the world [3,4], with suicide being the 4th leading cause of adolescent death in India [4]. Despite this, there remains a widely recognised lack of data on the prevalence of adolescent mental health in India, including anxiety and depression [5]. Additionally, much of the prevalence data for anxiety and depression among Indian adolescents is unclear and often inconsistent, which has been attributed to the use of limited data collection tools [5]. Culturally validated measures of youth mental health are critical for many reasons, from diagnostic accuracy and therapeutic impact to securing reliable prevalence rates to inform mental health policy and practice. However, globally, the limited number of culturally-appropriate mental health measures for young people is an obstacle in achieving this, with few accurately identifying symptoms of anxiety and depression cross-culturally [6]. A dearth of culturally-sensitive tools is seen particularly within lower-middle income countries, leaving large gaps in prevalence data and ways to monitor the impact of interventions [7]. This may partly be explained by most popular measures of depression and anxiety being developed in western countries, reducing their cross-cultural validity. Although there have been efforts to validate adolescent mental health measures in India [e.g., 8], this field remains limited. This study aimed to contribute to the metric-portfolio in India for generating reliable data on youth mental health. This study presents the first step in culturally validating and establishing the semantic equivalence a widely-used measure of child and youth mental health, namely the Revised Children’s Anxiety and Depression Scale [(RCADS; 9)].

### 1.1. The RCADS-47

The RCADS is a measure of symptoms of anxiety and depression for young people aged between 8 and 18-years. It is a 47-item self-report questionnaire containing six subscales; Separation Anxiety Disorder (SAD), Social Phobia (SoP), Obsessive-Compulsive Disorder (OCD), Panic Disorder (PD), Generalised Anxiety Disorder (GAD) and Major Depressive Disorder (MDD). The RCADS is used extensively worldwide [e.g., 6,9], has been validated for use in many countries [e.g., 10: Denmark.; 11: Australia] and shows good psychometric properties in several countries [(e.g., 9; 10)]. It is widely-used in India across diverse samples in both clinical and research settings, indicating that it is considered culturally and conceptually valid by experts [12]. Despite this extensive use, it has not yet been subjected to a thorough test of cultural validation for use within children or young people in India, with previous research suggesting that modifications may be needed to ensure applicability within this population [(e.g., 13)]. Previously Long et al. [8] psychometrically tested the unmodified RCADS with a sample of Indian adolescents, to establish factor structure, this study will build on this examining Indian adolescents’ interpretations of items to identify linguistic and cultural discrepancies, thereby ensuring the measure more accurately captures culturally grounded experiences of anxiety and depression.

### 1.2. The process of culturally validating a measure

Cross-cultural validation involves taking a measure which was designed originally for use in one culture, and showing it to be meaningful, applicable and equivalent within another culture [14]. The cultural validation of mental health measures is important as culturally validated measures can identify constructs that they have been designed to more accurately, when compared to measures which have not been culturally validated [15; 16].

The International Test Commission [17] presents guidelines for adapting and translating measures. This consists of 18 guidelines across six stages: (1**) pre-condition**; obtaining the necessary permission for the holders of intellectual rights of the selected measure, (2) **test development**; ensuing that the adaptation process considers linguistic, psychological and cultural differences and includes experts with relevant experience, (3) **confirmation**; empirical analysis of the adaptation in an appropriate sample (e.g., establishing reliability and validity), (4) **administration**; the preparation of administration material (e.g., testing guidelines) to minimise any cultural-related difficulties, (5) **score scales**; interpretation of any group score differences based on all available information and (6) **documentation**; provide technical documentation of any changes, including evidence obtained to support equivalence. As the beginning of the cultural validation of the RCADS-47, this study focuses on stages 1 and 2 of the ITC guidelines, pre-condition and test development.

This study also drew upon the cultural validation guidelines presented by Caron [18]. This states that cultural validation is achieved through establishing the validity and reliability of the measure in the new population and consists of two stages. Broadly, cultural validations of mental health measures are reflective of this and are often conducted in two stages, with the first stage involving the verification of the cultural equivalence of the measure [17–19], often in the form of a Think Aloud study, to determine ‘inferential equivalence’ within the target culture. This process is also informed by wider cross-cultural adaptation literature, including Flaherty et al. [20], who identified several key dimensions of cultural equivalence in psychological tools, including semantic, content, technical, criterion, and conceptual equivalence.Following this, psychometric testing is utilised to establish the measures validity and reliability in the target culture [17–19]. This study demonstrates Stage 1; verification of the cultural equivalence of the measure. This confirms that the measure maintains its ‘inferential equivalence’ within another culture, enabling it to accurately identify the constructs it was designed to. This is of particular importance within this validation study as the cultural conceptions and perceptions of mental health can differ between India and western countries [e.g., 21]. Cultural equivalence can be assessed by presenting the measure to a small sample of the target population to identify any misunderstandings of measure items and any changes that may be needed. However, it is acknowledged that this study does not constitute a full cultural validation or establish inferential equivalence. Rather, this study examines semantic equivalence, by assessing whether participants understood item wording as intended, and item equivalence, by exploring whether items were interpreted as relevant within participants’ lived contexts. In line with Flaherty et al. [20], this represents an initial exploration of semantic and content equivalence within the target population. The Think Aloud methodology also provided preliminary insight into participants’ response processes. These processes represent an important initial stage in the broader cultural adaptation and validation of the measure prior to psychometric testing.

Think Aloud interviews were selected as they are widely-used in research involving young people [e.g., 22–24] and are effective in determining young people’s cognitive processes when completing a standardised measure [25]. Due to the diversity of languages spoken in India [(121 legitimised languages; 26)], we tested the English-version of the RCADS. Think Aloud interviews have been used extensive within health research [(e.g., 27; 28)] and have been identified as a valuable tool for insight into participant understanding of measure items, leading to measure improvement [29]. This study presents the first stage of the cultural validation of the RCADS-47 within a sample of Indian adolescents.

Due to the large number of languages spoken in India [(121 legitimised languages; 26)], underlying religious connotation associated with different languages (e.g., Hindi is associated with Hinduism), and that in Karnataka young people are often taught in English in schools (with English also being a compulsory subject in all schools for Grade 5), validating the English version of the RCADS-47 was considered neutral and inclusive, alongside providing a good anchor language for further research. Per the 2011 census, English is the second most commonly spoken language in India, with the majority of these individuals speaking it as their second or third language (Ministry of Home Affairs India (2011).

### 1.3. The present study

This study presents the first stage (of two) of the cultural validation of the RCADS-47 within a sample of Indian adolescents aged between 14 and 17 years. The aim of this study was to examine the semantic, item, and response process equivalence of the RCADS-47 using cognitive interviewing methods with Indian adolescents, as a first stage towards its cultural validation. To achieve this objective, 12 Indian adolescents completed Think aloud interviews as they completed each item of the measure, enabling the and amendment of any potential cultural misunderstandings within the measure, informing any changes for Stage 2 of this cultural validation [(psychometric testing; 30)].

## 2. Method

### 2.1. Recruitment and Participants

Adolescents from a Youth Advisory Panel of an ongoing study were recruited [(Project SAMA; 31)], which is a collaboration between several UK and Indian universities and NIMHANS. The Youth Advisory Group was purposively recruited for the SAMA project based on their interest in adolescent mental health and their enthusiasm to shape research on this in India. Group members did not have to have experienced poor mental health themselves. They were indicative of the target population in terms of age, being school-going, from a mix of urban and rural locales and speaking English as well as local languages. Recruitment via this YPAG for the TA study was convenience sampling. The target sample was 10, as samples of five or more participants are adequate for Think Aloud studies [32] and is reflective of previous Think Aloud studies for measure analysis [(e.g., 23; 33)].

12 Indian adolescents aged between 14 and 17-years were recruited (5 males (41.7%), 7 females (58.3%); mean age = 15.67 years, *SD* = 0.98). The age group was selected as research indicates that adolescence is a critical time for the onset of mental health disorders [34]. Participants self-reported an overall English ability of 7.61 (cumulative of reading and speaking ability) on a scale of 1 (not well) to 10 (extremely well) and were taught in English in school, reducing the likelihood of problems encountered being due to English proficiency.

### 2.2. Ethics

All ethical safeguards were decided with NIMHANS and in-line with ethical guidelines in India [35]. The study was approved by the University of Leeds (PSYC-276) and NIMHANS ethics committee. Participants were encouraged to participate only if they were currently experiencing positive mental wellbeing at the point of consent. This criterion was adopted in part due to safeguarding considerations, as the study was not designed or resourced to appropriately support adolescents experiencing significant or clinical levels of distress, in line with ethical guidance for research with vulnerable populations [36; 37]. Although this may limit generalisability of findings, this study was intentionally focused on a non-clinical population. “*Positive emotional wellbeing*” was operationalised through adolescent self-identification at the point of recruitment. Participants and parents were provided, within the information sheet, with a brief and age-appropriate description of emotional wellbeing (e.g., generally feeling positive and not experiencing ongoing distress) and were advised to participate only if they felt this reflected their/ their child’s current experience. No formal screening tool was employed. Written parental and participant consent was obtained prior to the study, through online consent forms. Following initial written consent, participant consent was re-confirmed verbally at the start of the interview. Verbal consent was witnessed by the interviewer and audio recorded. Interviews were conducted between 21/09/2021 and 20/10/2021. Participants were reminded of their right to withdraw and given a handout containing sources of support for depression and anxiety, should they require them in the future.

### 2.3. Materials

The original 47-item English-version of the RCADS [9] was used. Thirty-seven items examine anxiety symptoms and ten examine depressive symptoms. Items are answered on a four-point scale depending on the frequency that the youth experiences the items from 0 (never) to 3 (always), producing a total internalising score (sum of all subscale), a total anxiety score (sum of the anxiety subscales) and a total depression score (sum of the depression subscale).

### 2.4. Procedure

Prior to commencement of the study, permission to use and culturally adapt the RCADS-47 was obtained from the copyright holders, consistent with Stage 1 of the ITC (2017) guidelines. Young people interested in participating were sent study information sheets for themselves and their parents. Parental and participant consent was obtained via an online written consent form. Participants also gave written consent for their data to be used in research via an online form. Following initial written consent, participant consent was re-confirmed verbally at the start of the interview, along with the collection of demographic information (Table 1). Verbal consent was witnessed by the interviewer and audio recorded. A unique ID code linked participant’s interview and demographic data. Both written and verbal instructions for the Think Aloud interviews were adapted from French et al. (2007). Participants completed an approximately 30-minute live, online audio-recorded Think Aloud interview with the first author (a UK national). An example of ‘thinking aloud’ was given to help participants familiarise themselves with the process During the interview, participants were asked to read and explain their comprehension of each item and their reason for their chosen response. Other than prompting the participant to continue talking if they fell silent for more than 10 seconds (‘keep thinking aloud’), the interviewer did not interrupt and remained off camera. Upon completion, participants were sent a debrief and a check on their wellbeing. This was deemed an appropriate length of interview for this measure based on cognitive interviewing research and practice, as this study aimed to identify how adolescents understand and respond to items rather than obtaining lengthy narrative focusing on comprehension, interpretation, and reasoning [38]. Research suggest that adequate responses can be obtained in this interview length [38]. Shorter interviews have also been shown to reduce fatigue and help maintain adolescents’ engagement and concentration [39]. Audio-recordings were transcribed verbatim.

**Table 1:** Participant demographic information.

*Demographic*	Response	N	Percentage (%)
School Type (SES)	Private school (unaided)	10	83.3
	Government aided private school	1	8.3
	Other	1	8.3
Parental Education (SES)	Master’s Degree	3	25
	Bachelor’s Degree	2	26.7
	Higher secondary school certificate	4	33.3
	Secondary School certificate	1	8.3
	Not sure	2	16.7
Parental Work (SES)	Two parents working full time	4	33.3
	One parent working full time	4	33.3
	One parent working full time and one parent working part time	1	8.3
	Two parents working part time	1	8.3
	Prefer not to say	2	16.7
Language	Kannada	5	41.7
	Kannada and English	1	8.3
	Lambani	1	8.3
	Tamil	1	8.3
	Telugu	4	33.3
Location	Rural	2	16.7
	Urban	7	58.3
	Semi-Urban	2	16.7
	Not sure	1	8.3

### 2.5. Analysis

Analysis of transcripts was completed by the first author using a method adopted from previous Think Aloud studies [40; 41]. This involved coding each interview segment into at least one of six coding categories (Table 2), and if needed, more than one. Categories were; (1) no problem; (2) no sufficient thinking aloud; (3) reread/ stumbled; (4) problems understanding and (5) misinterpretation of question. As participants completed the interviews in a language which was not their first language, and as several responses did not fall into any of the pre-existing categories, a new coding category was added, namely (6) ‘responses did not make sense in English’. Consistent with recommendations for cognitive interviewing and Think Aloud methodology in questionnaire adaptation research, the frequency and type of identified comprehension and interpretation issues were reported to indicate the relative prominence of item-level difficulties within the sample, rather than to imply statistical generalisability [43; 44].

**Table 2:** Coding categories for the Think Aloud interviews.

Category	Description
1.No Problem	There were no significant problems identified.
2. No sufficient thinking aloud	Participants did not report sufficient information for coding purposes on any of the four cognitive processes [42].
3. Reread/stumbled	Participants re-read a question or seriously stumbled (e.g., stammered/stuttered/significantly paused) whilst reading it. Participants re-reading a question does not necessarily mean that they had problems in understanding the question, more than one participant re-reading a question may indicate this question requires efforts to understand.
4. Problems understanding	Participants showed that they experienced difficulty when understanding or answering the question. For example, they stated that they would need more information before they could answer it, appeared confused by the question, questioned the meaning of the question or stated that they were unsure if they had answered or understood the question properly.
5. Misinterpretation of Question	The participant appeared to answer a different question to the one that was asked, or gave reasoning which seemed to be inconsistent with, or irrelevant to, the answer that was given.
6. Response did not make sense in English	The participant answered the question however their response did not make sense in English. This does not necessarily indicate that the participant had problems in understanding the question and instead is likely to be caused by an error in translation when verbalising the response, due to English not being their first language. Multiple participant responses not making sense in English may indicate problems understanding the question.

After full initial coding, 53% of item responses were randomly selected for blind second coding, yielding a good level of agreement [(83.33% agreement; K = 0.75; 45)]. Disagreements were resolved through discussion and coding was amended accordingly. Once coding was established, problematic items on the RCADS were identified and suggested rephrasing was developed. Changes were developed with guidance from Indian external advisors, for example, where difficulties in comprehension were identified, these terms were subsequently discussed with the Indian research team to identify more culturally and linguistically appropriate alternatives that would be more commonly understood within the target population.

As a general rule, we adopted a 25% cut-off, with items generating 3 or more problems (i.e., at least three participants having trouble with that item across any of the six coding categories) being candidates for rephrasing, similar to previous Think Aloud studies [(e.g., 24)]. Consistent with Step 2 of the ITC adaptation process, bilingual in-country mental health professionals from the SAMA project were involved throughout the adaptation process. Their expertise informed the interpretation of Think Aloud data, identification of potential semantic and cultural issues within items, and discussions regarding alternative phrasing to improve clarity and contextual relevance for adolescents in Karnataka. This expert input complemented the participant-based assessment of semantic and item equivalence presented in the current study.

## 3. Results

Twelve participants responded to the 47 items of the RCADS, generating 564 (12 × 47) segments of interview text. Of the 564 segments, 158 (28%) were assigned exclusively to category 2 (No sufficient thinking aloud) and were removed from the analysis, leaving 406 segments. Of these, 386 (95.07%) were assigned to one coding category and 20 (4.93%) were assigned to two categories.

The coding of the remaining 406 segments identified a total of 97 problems relating to 33 RCADS items (see Table 3). This threshold was selected to balance sensitivity and specificity, ensuring that item revision was driven by systematic patterns of difficulty rather than isolated or idiosyncratic misunderstandings. Within Cognitive Interviewing, there is no universally prescribed numerical criterion; however, methodological guidance emphasises the importance of identifying recurrent response problems across participants as evidence of substantive threats to Response Process Validity [(e.g., 43,46]. Given typical sample sizes in think-aloud studies (often 10–15 participants), a threshold of three or more participants has been recommended as indicating meaningful and non-trivial comprehension or interpretation issues (e.g., [47]). Thus, the 25% criterion provides a pragmatic and empirically grounded approach to identifying problematic items while avoiding over-modification based on limited evidence.

**Table 3:** The frequency and the category of the problems identified for each item of the RCADS-47.

Construct (N items)	Items	Total N problems	Reread/ stumbled	Problems understanding	Misinter-pretation	Not make sense in English
**MDD (10)**	2. I feel sad or empty.	2	1	–	–	1
	6. Nothing is much fun anymore.	3	1	1	1	–
	15. I have problems with my appetite	5	3	1	1	–
	21. I am tired a lot	3	3	–	–	–
	25. I cannot think clearly	2	1	–	–	1
	29. I feel worthless	1	1	–	–	–
	40. I feel like I don’t want to move	5	2	2	1	–
	47. I feel restless	3	–	–	3	–
	***MDD Total***	***24***	***12***	***4***	***6***	***2***
**GAD (6)**	1. I worry about things	3	1	–	–	2
	13. I worry that something awful will happen to someone in my family	3	1	1	1	–
	27. I worry that something bad will happen to me	3	2	–	1	–
	35. I worry about what is going to happen	3	2	–	–	1
	***GAD Total***	***12***	***6***	***1***	***2***	***3***
**PD (9)**	3. when I have a problem I get a funny feeling in my stomach	4	1	1	2	–
	14. I suddenly feel as if I can’t breathe when there is no reason for this	5	1	2	2	–
	28. When I have a problem I feel shaky	1	1	–	–	–
	34. All of a sudden I feel really scared for no reason at all	1	1	–	–	–
	36. I suddenly feel faint or dizzy when there is no reason for this	2	1	–	1	–
	39. My heart suddenly starts to beat too quickly for no reason	2	1	–	–	1
	41. I worry that I will suddenly get a scared feeling when there is nothing to be afraid of	3	1	–	–	2
	***PD Total***	***18***	***7***	***3***	***5***	***3***
**SoP (9)**	4. I worry when I think I have done poorly at something	3	1	–	2	–
	7. I feel scared when I have to take a test	2	1	–	1	–
	12. I worry that I will do badly at my school work	1	1	–	–	–
	20. I worry I might look foolish	1	–	–	–	1
	43. I feel afraid that I will make a fool of myself in front of other people	2	–	–	–	2
	***SoP Total***	***9***	***3***	***0***	***3***	***3***
**SAD (7)**	5. I would feel afraid of being on my own at home	2	1	–	–	1
	17. I feel scared if I have to sleep on my own	2	2	–	–	–
	33. I am afraid of being in crowded places (like shopping centres, the movies, busses, busy playgrounds)	1	1	–	–	
	46. I would feel scared if I had to stay away from home overnight	2	2	–	–	–
	***SAD Total***	***7***	***6***	***0***	***0***	***1***
**OCD (6)**	16. I have to keep checking that I have done things right (like the switch is off, or the door is locked)	7	2	–	4	1
	23. I can’t seem to get bad or silly thoughts out of my head	2	2	–	–	–
	31. I have to think of special thoughts (like numbers or words) to stop bad things from happening	4	1	1	2	–
	42. I have to do some things over and over again (like washing my hands, cleaning or putting things in a certain order)	7	1	–	6	–
	44. I have to do some things in just the way to stop bad things from happening	7	2	–	5	–
	***OCD Total***	***27***	***8***	***1***	***17***	***1***
***Total***	***RCADS Total***	***97***	***42***	***9***	***33***	***13***

Eighteen items generated a total number of problems of 3 or higher (range: 3–7). However, each item problem was assessed individually before a rephrasing decision was made. Examples of problems experienced by participants are presented in Appendix A. We identified 14 items which were problematic and in need of rephrasing. Suggested rephrasing is shown in Table 4; most changes are minor modifications to wording to clarify item meaning. The Hindi RCADS was not consulted as this study focused on semantic understanding of the English version among adolescents in Karnataka, which would have been obscured by relying on an already translated adaptation. Instead, in line with cultural adaptation guidelines, item interpretation was grounded directly in participants’ response processes [17,19].

**Table 4:** Suggested rephrasing for problematic items of the RCADS.

RCADS Subscale	RCADS item	Suggested rephrasing	Rationale for rephrasing
MDD	6. Nothing is much fun anymore	I do not have a lot of fun anymore	Phrasing not commonly used when speaking English in India.
	15. I have problems with my appetite	I have problems with my eating patterns	Appetite is not commonly used when speaking English in India.
	21. I am tired a lot	I feel tired a lot of the time	Phrasing not commonly used when speaking English in India.
	40. I feel like I don’t want to move	I feel like I don’t have energy to move	Further explanation needed to increase clarity.
	47. I feel restless	I feel restless and it is hard for me to sit still or relax	Further explanation needed to increase clarity.
GAD	13. I worry that something awful will happen to someone in my family	I worry that something bad will happen to someone in my family (e.g., my parents, brother or sister)	Phrasing not commonly used when speaking English in India and further explanation was needed due to different family structures.
PD	3. When I have a problem I get a funny feeling in my stomach	When I have a problem, my stomach doesn’t feel good	Phrasing not commonly used when speaking English in India.
	14. I suddenly feel as if I can’t breathe when there is no reason for this	I suddenly feel it is difficult to breathe even though there is no clear reason for this	Phrasing not commonly used when speaking English in India.
	41. I worry that I will suddenly get a scared feeling when there is nothing to be afraid of	I worry that I will suddenly feel scared even though there is nothing to be afraid of	Phrasing not commonly used when speaking English in India.
SoP	4. I worry when I think I have done poorly at something	I worry when I think I have done badly at something	‘Poorly’ is not commonly used when speaking English in India.
OCD	16. I have to keep checking that I have done things right (like the switch is off, or the door is locked)	I spend a lot of time checking things repeatedly even when there is no need (e.g., if the door is locked)	Phrasing not commonly used when speaking English in India; confusion surrounding ‘done things right’; example not applicable in India due to energy shortages.
	31. I have to think of special thoughts (like numbers or words) to stop bad things from happening	I need to use special thoughts (like numbers or words) to stop bad things from happening	Phrasing not commonly used when speaking English in India.
	42. I have to do some things over and over again (like washing my hands, cleaning or putting things in a certain order)	I have to do some things repeatedly to feel okay (e.g., putting things in a certain order)	Phrasing not commonly used when speaking English in India; example not applicable in India due to religious norms.
	44. I have to do some things in just the way to stop bad things from happening	I sometimes think that bad things will happen, to stop the bad things from happening I have to do some things in just the right way	Phrasing not commonly used when speaking English in India.

Overall, participants re-reading the question or stumbling while reading the question (*n* = 42; Category 3) and misinterpretation of the question (*n* = 33; Category 5) were identified as the most commonly occurring problems. Multiple participants re-reading or stumbling while reading an item is an important indicator that the item requires effort for participants understand [40], potentially indicating misunderstanding or confusion. When completing a measure, it is as important that items are easy to read and understand as it is for them to be conceptually valid.

### 3.1. Obsessive-Compulsive Disorder Subscale

The OCD subscale yielded the most problems (*n* = 27), with 4 of the 6 items being identified for rephrasing. A range of problems were encountered by participants in this subscale, with the most common being misinterpretation of the item (*n* = 17). Participants struggled with several aspects of the OCD items, with many failing to identify the obsessive repetitive element, instead responding as if the item was relating to aspects of their day-to-day life. For example, in response to item 16 (‘*I have to keep checking that I have done things right (like the switch is off, or the door is locked’*)), many participants did not identify that this item was addressing an obsessive need to continuously ensure that things have been done correctly, and instead referred to everyday tasks that have to be completed or checked (e.g., household chores). Additionally, participants struggled to understand the internal or self-focusing nature of items, and instead responses centred around societal or family expectations. For example, in response to item 44 (‘*I have to do some things in just the way to stop bad things from happening’*), instead of focusing on an internal drive to do things in a certain way, several participants discussed societal actions or changes, with one participants discussing studying social work to combat this.

### 3.2. Major Depressive Disorder Subscale

The MDD subscale yielded the second highest number of problems (*n* = 24), with the most commonly identified problem being participants re-reading/ stumbling, indicating that the participants expended notable ‘effort’ while understanding the item (*n* = 12). Overall, 5 of the 10 MDD items were identified for rephrasing. However, these suggested changes mostly consisted of minor modifications to wording, with limited significant problems surrounding understanding being identified. Analysis suggests these problems may be caused by items being worded in a complicated way, or including phrases which are uncommon in India. For example, three participants showed difficulty in reading item 15 (‘*I have problems with my appetite’*). It was confirmed by our external advisors that alternative phrasing, such as ‘eating patterns’, may aid understanding. This is further observed in item 6 (‘*Nothing is much fun anymore’),* where the word ‘much’ has been identified as not being commonly used in this context in India.

### 3.3. Panic Disorder, Generalised Anxiety Disorder and Social Phobia Subscales

Several problems were identified in the PD (N = 18), GAD (N = 12) and SoP (*n* = 9) subscales, with 3 PD items, 1 GAD items and 1 SoP item being identified for rephrasing. All subscales identified re-reading/ stumbling as a main problem, indicating some items required significant effort to understand. Items within these subscales were identified as including complicated language or language not commonly used in India, for example item 3 (*‘when I have a problem I get a funny feeling in my stomach’*), with the phrase ‘a funny feeling in my stomach’ proving difficult for our participants to understand.

## 4. Discussion

This study aimed to examine the semantic, item, and response process equivalence of the RCADS-47 using cognitive interviewing methods with Indian adolescents, as a first stage towards its cultural validation and presents the first of two stages towards the cultural validation of the RCDAS-47 in a sample of Indian adolescents aged 13–17 years in Karnataka. Of the 47 items, 14 items were identified as problematic.

This finding was expected as research indicates that few anxiety and depression measures are cross-culturally valid [6]. Items yielding three or more problems were identified as in need of rephrasing. Overall, participants re-reading the question or stumbling while reading the question (*n* = 42; Category 3) and misinterpretation of the question (*n* = 33; Category 5) were the most common problems. The OCD subscale yielded the most problems (*n* = 27), with four of the six items being identified for rephrasing, followed by the Major Depressive Disorder subscale (*n* = 12), with five of the 10 Major Depressive Disorder items being problematic. There are several potential explanations for these problems.

First, research indicates that symptoms of Obsessive-Compulsive Disorder can vary cross-culturally, with differences in symptom content, expression, and severity reported between non-Western and Western populations [e.g., 48; 49]. Studies conducted in Indian samples have identified distinct clinical presentations, including greater prominence of religious and contamination-related obsessions, as well as culturally embedded compulsive behaviours [49]. As the RCADS was developed in the United States based on Western conceptualisations of OCD, some item content may not adequately reflect culturally specific manifestations of symptoms in Indian adolescents. For example, repetitive cleaning behaviours (e.g., Item 42) are commonly interpreted as pathological within Western diagnostic frameworks; however, in the Indian context, practices related to cleanliness are often embedded within religious and cultural norms, particularly within Hinduism [50]. Empirical research further suggests that higher levels of religiosity are associated with increased endorsement of OCD-like traits [51], raising the possibility that such items may conflate culturally normative behaviours with psychopathology in certain populations. While the relationship between religiosity and OCD is complex and multifaceted [51], these findings highlight potential challenges in the cross-cultural validity of OCD subscales. Additionally, low mental health literacy regarding OCD among Indian adolescents [52] may contribute to misunderstandings of item content, potentially affecting response accuracy. Finally, the OCD subscale itself remains contentious, both within the RCADS and more broadly, as reflected in its reclassification outside of anxiety disorders in the DSM-5 and ongoing discussions regarding its inclusion in the RCADS [e.g., 53]. Although the OCD subscale was a prominent focus within the findings, OCD is no longer classified as an anxiety disorder within the DSM-5, despite its inclusion in the original RCADS framework at the time of development. Therefore, the OCD findings should be interpreted cautiously.

Additionally, as data was collected following the COVID-19 pandemic, it must be considered that COVID-19 and resulting government policies brought about a change in behavioural norms not present when the RCADS was developed. For example, Item 42 gives repetitive handwashing as an example of OCD; however, several participants discussed that they now did this regularly, with one specifically stating that frequent hand washing was normal during the pandemic. Therefore, presently, repetitive handwashing is not a suitable example of an OCD symptom. This raises questions as to whether all OCD measures should be revised to account for the impact of the pandemic on behavioural norms.

Second, difficulties may be due to item phrasing not being common in Indian English. This finding was observed in all subscales. Additionally, English language proficiency also influences comprehension and complex words or phrases may be difficult for those with low English language abilities. This has been observed in previous research using the RCADS where Indian adolescents struggled to understand item meaning in the OCD subscale [54]. This problem was particularly evident within the MDD subscale. This difficulty may reflect cultural and linguistic differences in how depressive symptoms are conceptualised and expressed among Indian adolescents. Psychological terminology, as well as commonly used Western terms, may not translate directly across cultures, particularly for items describing internal or somatic experiences [19,20]. For example, terms such as “appetite” (Item 15) may be less commonly used or interpreted differently within everyday adolescent language in the Indian context. This is particularly relevant for the MDD subscale, where depressive symptoms often include somatic and internally experienced states that may be described differently across cultures, potentially affecting how items are understood and endorsed.

This extends to the PD subscale, which may reflect cultural differences in how acute anxiety is experienced and labelled, with symptoms often expressed in somatic or contextual terms rather than as “panic” in a clinical sense, such as dizziness, breathlessness etc. [55]. This, alongside limited familiarity with diagnostic terminology, may lead to misunderstanding of item wording during. The numerous problems identified within these subscales relating to language means we should be cautious in relying on research using the culturally un-validated RCADS in India. Therefore, we propose that that problematic RCADS items be rephrased to remove culturally-bound phrasing and examples. This study has generated a simpler alternative wording commonly used in India, alongside additional explanations for potentially complex items, with the aid of external advisors in India.

### 4.1. Strengths, limitations and future research

The strengths of this study include the thorough approach towards measure validation. Some validation studies do not include this first stage and only conduct psychometric analysis, but this can limit the resulting measure as linguistic cultural equivalence has not been established.

However, findings should be considered in view of several limitations. Our sample is clearly not representative, nor aimed to be, of all young people in India. For example, the majority of participants in this study reported that they were Hindu, as discussed above, religion and religious practices may have some influence over daily norms and therefore experiences of anxiety symptoms [e.g., 51; 56], potentially influencing young people’s interpretation of the items. Therefore, although Hinduism is the prominent religion in India, findings between young people of different religions may differ (e.g., Muslims who make up 14.2% of the population (Ministry of Home Affairs India (2011)).

More broadly, India is highly culturally, linguistically, and socioeconomically diverse, and understandings, expressions, and communication of mental health symptoms may vary substantially across regions, communities, family structures, and social groups [e.g., 21]. Cultural norms relating to emotional expression, family relationships, academic expectations, social responsibility, and stigma surrounding mental health may all influence how adolescents interpret questionnaire items and decide upon responses. Consequently, certain RCADS items may hold different meanings, levels of relevance, or emotional salience in different Indian contexts. Although the current study provides preliminary evidence of semantic and item equivalence among adolescents in Karnataka, further work is needed to explore whether similar interpretations are observed across adolescents from different linguistic, religious, rural/urban, and socioeconomic backgrounds within India.

Additionally, all participants were from Karnataka. Although it has been verified by in-country advisors that young people in Karnataka do not speak a particularly unique version of Indian-English, suggesting that difficulties experienced by young people in Karnataka would be similar to young people from different states, some linguistic difference may still exist. Despite this, standardised English is taught in schools throughout India, suggesting that all school-going young people should have a similar understanding of standardised English, however findings should be applied to all areas of India with caution, due to potential linguistic differences that may exist.

Participants were recruited on the basis of self-identified positive emotional wellbeing, which may have introduced selection bias and restricts the generalisability of the findings. As a result, the perspectives captured are likely to reflect those of adolescents who are currently functioning well, and may not represent those experiencing emotional distress or clinical levels of difficulty.

Furthermore, participants were mostly from high Socio-Economic Status (SES) backgrounds and living in urban areas. This homogeneity may be partly due to the study being conducted online during COVID-19 travel restrictions, resulting in a sample consisting solely of adolescents with internet access. Consequently, adolescents from lower SES backgrounds were underrepresented, substantially limiting the generalizability of findings to the broader adolescent population in Karnataka and India more widely. This limitation is particularly important given the well-documented digital divide in India, where internet access differs significantly according to socioeconomic status and rural–urban location [57]. Adolescents from urban, high-SES backgrounds may also have had greater exposure to English and higher educational opportunities, potentially reducing the identification of language and comprehension difficulties within the RCADS items. Previous research has shown that digital exclusion and educational inequalities may disproportionately affect rural adolescents and those from disadvantaged backgrounds [57]. Therefore, future validation studies should prioritize recruiting adolescents from rural and lower-SES populations to improve representativeness and cultural applicability of the RCADS-47. A further limitation is that, although this study is a step towards validating the RCADS-47 in adolescents aged 13–17 years, no 13-year-olds were recruited at this stage of the study.

A further limitation of the study may be that participants were recruited through the SAMA Youth Advisory Panel, whose members had a pre-existing interest in adolescent mental health and research involvement. Although the group was broadly reflective of the target population in terms of age, school attendance, geographic background, and language use, participants may have been more familiar with mental health concepts and terminology than adolescents in the wider population. As such, the study may underestimate potential difficulties in understanding certain items or the broader concept of mental health measurement among adolescents with less exposure to these topics.

The absence of 13-year-old participants is a limitation as it reduces the representativeness of the sample across the full 13–17 age range and limits generalisability of findings to younger adolescents. No 13-year-olds were included in this study as none opted in to take part, despite the study being advertised to this age group. This is important because younger adolescents may differ in cognitive and linguistic development, which can affect how they interpret and articulate responses during Think Aloud tasks [58]. Their non-participation may reflect lower consent uptake or engagement among younger adolescents, a common issue in adolescent mental health research [59].

Finally, individual differences may influence a young person’s ability to think aloud [(e.g., 60; 61)], however, individual differences while thinking aloud are more prominent during tasks which cause a high cognitive load [(e.g., manually completing a task while thinking aloud; 61)], which was not present in our study. This was also addressed by including a relatively high number of participants.

Future research should aim to conduct think aloud interviews exploring the RCADS-47 with more diverse samples, this will enable any sub-cultural or demographic differences of item understanding to be identified. Identifications of further problems in different demographics will enable the RCADS-47 to be adapted further. Finally, the rephrased RCADS-47, as presented in this study, should be psychometrically tested in a large sample of Indian adolescents. If statistical testing reveals that the measure has met the acceptable criteria for the appropriate tests, cultural validity will have been established [18], increasing confidence in the use of the RCADS among Indian adolescents.

## 5. Conclusion

Overall, a wide-range of problems were encountered by a sample of Indian adolescents when completing the English-version of the RCADS-47. We suggest that 14 items should be rephrased to increase the cultural equivalence of the RCADS-47. To strengthen support for our findings and confirm the cultural validity of the RACDS-47 among Indian adolescents, further research, establishing validity and reliability of the RCADS with the rephrased items, should be conducted. A second stage of the validation process will involve psychometric evaluation of the revised measure within a larger sample of Indian adolescents. This will include examination of the reliability and construct validity of the adapted RCADS, including assessment of internal consistency, convergent and discriminant validity, and factor structure. The aim of this subsequent stage will be to determine whether the culturally adapted measure demonstrates appropriate psychometric properties and retains the intended anxiety and depression constructs within the Indian context

## Supporting information

S1 Appendix Examples of a selection of problems experienced by participants when responding to the RCADS items. (DOCX)

## References

[1] Gururaj G, Varghese M, Benegal V, Rao GN, Pathak K, Singh LK. National Mental Health Survey of India, 2015–16. Bengaluru: NIMHANS Publication. 2016.

[2] Aggarwal S, Berk M. Evolution of adolescent mental health in a rapidly changing socioeconomic environment: a review of mental health studies in adolescents in India over last 10 years. Asian J Psychiatr. 2015;13:3–12. doi: 10.1016/j.ajp.2014.11.007 25499463

[3] Patel V, Ramasundarahettige C, Vijayakumar L, Thakur JS, Gajalakshmi V, Gururaj G, et al. Suicide mortality in India: a nationally representative survey. Lancet. 2012;379(9834):2343–51. doi: 10.1016/S0140-6736(12)60606-0 22726517 PMC4247159

[4] Senapati RE, Jena S, Parida J, Panda A, Patra PK, Pati S, et al. The patterns, trends and major risk factors of suicide among Indian adolescents – a scoping review. BMC Psychiatry. 2024;24(1):35. doi: 10.1186/s12888-023-05447-8 38195413 PMC10775453

[5] Grover S, Raju VV, Sharma A, Shah R. Depression in Children and Adolescents: A Review of Indian studies. Indian J Psychol Med. 2019;41(3):216–27. doi: 10.4103/IJPSYM.IJPSYM_5_19 31142922 PMC6532377

[6] Stevanovic D, Bagheri Z, Atilola O, Vostanis P, Stupar D, Moreira P, et al. Cross-cultural measurement invariance of the Revised Child Anxiety and Depression Scale across 11 world-wide societies. Epidemiol Psychiatr Sci. 2017;26(4):430–40. doi: 10.1017/S204579601600038X 27353487 PMC6998552

[7] Erskine HE, Enright ME, Blondell SJ, Shadid J, Scott JG, Whiteford HA. Core Principles of International Research: Lessons From the National Adolescent Mental Health Surveys. Journal of Adolescent Health. 2023;72(1):S15–7. doi: 10.1016/j.jadohealth.2021.05.006 36229398

[8] Long KNG, Long PM, Pinto S, Crookston BT, Gren LH, Mihalopoulos NL, et al. Development and validation of the Indian Adolescent Health Questionnaire. J Trop Pediatr. 2013;59(3):231–42. doi: 10.1093/tropej/fmt006 23418132 PMC3693506

[9] Chorpita BF, Yim L, Moffitt C, Umemoto LA, Francis SE. Assessment of symptoms of DSM-IV anxiety and depression in children: a revised child anxiety and depression scale. Behav Res Ther. 2000;38(8):835–55. doi: 10.1016/s0005-7967(99)00130-8 10937431

[10] Esbjørn BH, Sømhovd MJ, Turnstedt C, Reinholdt-Dunne ML. Assessing the Revised Child Anxiety and Depression Scale (RCADS) in a national sample of Danish youth aged 8-16 years. PLoS One. 2012;7(5):e37339. doi: 10.1371/journal.pone.0037339 22649520 PMC3359357

[11] de Ross RL, Gullone E, Chorpita BF. The Revised Child Anxiety and Depression Scale: A Psychometric Investigation with Australian Youth. Behav change. 2002;19(2):90–101. doi: 10.1375/bech.19.2.90

[12] Trevethan M, Jain AT, Shatiyaseelan A, Luebbe AM, Raval VV. A longitudinal examination of the relation between academic stress and anxiety symptoms among adolescents in India: The role of physiological hyperarousal and social acceptance. Int J Psychol. 2022;57(3):401–10. doi: 10.1002/ijop.12825 34928515

[13] Raval VV, Luebbe AM, Sathiyaseelan A. Parental socialization of positive affect, adolescent positive affect regulation, and adolescent girls’ depression in India. Social Development. 2018;28(2):274–89. doi: 10.1111/sode.12325

[14] Matsumoto D, Davis SF. Handbook of research methods in experimental psychology. In: Davis SF, editor. Cross-cultural research. Oxford: Blackwell. 2003.

[15] Ali G-C, Ryan G, De Silva MJ. Validated Screening Tools for Common Mental Disorders in Low and Middle Income Countries: A Systematic Review. PLoS One. 2016;11(6):e0156939. doi: 10.1371/journal.pone.0156939 27310297 PMC4911088

[16] Weobong B, Akpalu B, Doku V, Owusu-Agyei S, Hurt L, Kirkwood B, et al. The comparative validity of screening scales for postnatal common mental disorder in Kintampo, Ghana. J Affect Disord. 2009;113(1–2):109–17. doi: 10.1016/j.jad.2008.05.009 18614241

[17] International Test Commission. The ITC Guidelines for Translating and Adapting Tests (Second Edition). 2017. http://www.InTestCom.org

[18] Caron J. A guide for cross-cultural validation of measurement instruments in mental health. 1999. doi: 10.13140/RG.2.1.3730.5685

[19] Beaton DE, Bombardier C, Guillemin F, Ferraz MB. Guidelines for the process of cross-cultural adaptation of self-report measures. Spine (Phila Pa 1976). 2000;25(24):3186–91. doi: 10.1097/00007632-200012150-00014 11124735

[20] Flaherty JA, Gaviria FM, Pathak D, Mitchell T, Wintrob R, Richman JA, et al. Developing instruments for cross-cultural psychiatric research. J Nerv Ment Dis. 1988;176(5):257–63. doi: 10.1097/00005053-198805000-00001 3367140

[21] Gaiha SM, Taylor Salisbury T, Koschorke M, Raman U, Petticrew M. Stigma associated with mental health problems among young people in India: a systematic review of magnitude, manifestations and recommendations. BMC Psychiatry. 2020;20(1):538. doi: 10.1186/s12888-020-02937-x 33198678 PMC7667785

[22] Davies EL, Martin J, Foxcroft DR. Development and Acceptability of a Co-Produced Online Intervention to Prevent Alcohol Misuse in Adolescents: A Think Aloud Study. JMIR Hum Factors. 2015;2(2):e13. doi: 10.2196/humanfactors.4452 27025403 PMC4797700

[23] Cho B-Y. Competent Adolescent Readers’ Use of Internet Reading Strategies: A Think-Aloud Study. Cognition and Instruction. 2014;32(3):253–89. doi: 10.1080/07370008.2014.918133

[24] Zhang Q, Hugh-Jones S, O’Connor DB. Investigation of psychometric properties of the Mindful Eating Questionnaire in Chinese adolescents and young adults using mixed methods. Appetite. 2022;176:106097. doi: 10.1016/j.appet.2022.106097 35654223

[25] Yardley L, Morrison LG, Andreou P, Joseph J, Little P. Understanding reactions to an internet-delivered health-care intervention: accommodating user preferences for information provision. BMC Med Inform Decis Mak. 2010;10:52. doi: 10.1186/1472-6947-10-52 20849599 PMC2946266

[26] Jolad S, Agarwal A. Mapping India’s language and mother tongue diversity and its exclusion in the Indian census. 2021. doi: 10.31235/osf.io/sjxc6

[27] Galasinski D. Men’s discourses of depression. New York, NY: Palgrave Macmillan. 2008.

[28] Mallinson S. Listening to respondents: a qualitative assessment of the Short-Form 36 Health Status Questionnaire. Soc Sci Med. 2002;54(1):11–21. doi: 10.1016/s0277-9536(01)00003-x 11820675

[29] Phillips AC. The Usefulness of ‘think-aloud ‘for Evaluating Questionnaires in Use in the Health Domain. United Kingdom: The University of Manchester. 2014.

[30] PalmerA, O’ConnorDB, JanardhanaN, KhandeparkarP, BholaP, PrabhuS, et al. Toward cultural validation of the revised children’s anxiety and depression scale in Karnataka, India: Psychometric testing among 13–17‐year olds. Mental Health Science. 2025;3(2):e70019.10.1371/journal.pone.035270542616736

[31] Hugh-Jones S, Janardhana N, Al-Janabi H, Bhola P, Cooke P, Fazel M, et al. Safeguarding adolescent mental health in India (SAMA): study protocol for codesign and feasibility study of a school systems intervention targeting adolescent anxiety and depression in India. BMJ Open. 2022;12(4):e054897. doi: 10.1136/bmjopen-2021-054897 35379625 PMC8981280

[32] Nielsen J. Estimating the number of subjects needed for a thinking aloud test. International Journal of Human-Computer Studies. 1994;41(3):385–97. doi: 10.1006/ijhc.1994.1065

[33] Lau K. Reading strategy use between Chinese good and poor readers: a think‐aloud study. J Res Reading. 2006;29(4):383–99. doi: 10.1111/j.1467-9817.2006.00302.x

[34] Jones PB. Adult mental health disorders and their age at onset. Br J Psychiatry Suppl. 2013;54:s5-10. doi: 10.1192/bjp.bp.112.119164 23288502

[35] I C M R. National Ethical Guidelines for Biomedical and Health Research Involving Human Participants. 2017. https://www.icmr.nic.in/guidelines/ICMR_Ethical_Guidelines_2017.pdf 10.20529/IJME.2018.065 30211674

[36] Oates J, Carpenter D, Fisher M, Goodson S, Hannah B, Kwiatowski R, et al. BPS code of human research ethics. British Psychological Society. 2021.

[37] World Health Organization. Standards and operational guidance for ethics review of health-related research with human participants. World Health Organization. 2011. 26269877

[38] Ericsson KA, Simon HA. Protocol analysis: verbal reports as data. Rev. ed. ed. MIT Press. 1993.

[39] Young JP, Schraedley PK. Investigating the information-seeking processes of adolescents: The value of using think alouds and think afters. Library & Information Science Research. 2000;22(4):371–92.

[40] French DP, Cooke R, McLean N, Williams M, Sutton S. What do people think about when they answer theory of planned behaviour questionnaires? A “think aloud” study. J Health Psychol. 2007;12(4):672–87. doi: 10.1177/1359105307078174 17584818

[41] van Oort L, Schröder C, French DP. What do people think about when they answer the Brief Illness Perception Questionnaire? A “think-aloud” study. Br J Health Psychol. 2011;16(Pt 2):231–45. doi: 10.1348/135910710X500819 21489052

[42] Tourangeau R, Rips LJ, Rasinski K. The psychology of survey response. 2000.

[43] Beatty PC, Willis GB. Research Synthesis: The Practice of Cognitive Interviewing. Public Opinion Quarterly. 2007;71(2):287–311. doi: 10.1093/poq/nfm006

[44] Willis GB, Miller K. Cross-cultural cognitive interviewing: Seeking comparability and enhancing understanding. Field Methods. 2011;23(4):331–41. doi: 10.1177/1525822X11416092

[45] McHugh ML. Interrater reliability: the kappa statistic. Biochem Med (Zagreb). 2012;22(3):276–82. 23092060 PMC3900052

[46] Willis GB. Cognitive interviewing: A tool for improving questionnaire design. sage publications. 2005.

[47] Blair J, Conrad FG. Sample size for cognitive interview pretesting. Public Opin Q. 2011;75(4):636-58. doi: 10.1093/poq/nfr035

[48] Nicolini H, Salin-Pascual R, Cabrera B, Lanzagorta N. Influence of Culture in Obsessive-compulsive Disorder and Its Treatment. Curr Psychiatry Rev. 2017;13(4):285–92. doi: 10.2174/2211556007666180115105935 29657563 PMC5872369

[49] Sharma E, Tripathi A, Grover S, Avasthi A, Dan A, Srivastava C, et al. Clinical profile of obsessive-compulsive disorder in children and adolescents: A multicentric study from India. Indian J Psychiatry. 2019;61(6):564–71. doi: 10.4103/psychiatry.IndianJPsychiatry_128_19 31896861 PMC6862976

[50] Dhand A. The Dharma of Ethics, the Ethics of Dharma: Quizzing the Ideals of Hinduism. Journal of Religious Ethics. 2002;30(3):347–72. doi: 10.1111/1467-9795.00113

[51] Agorastos A, Cüney D, Huber Christian G. Influence of religious aspects and personal beliefs on psychological behavior: focus on anxiety disorders. Psychology Res Behavior Manag. 2014;:93–101. 10.2147/PRBM.S43666 PMC3956626 24648780

[52] Ahmad A, Salve HR, Nongkynrih B, Sagar R, Krishnan A. Mental health literacy among adolescents: Evidence from a community-based study in Delhi. Int J Soc Psychiatry. 2022;68(4):791–7. doi: 10.1177/00207640211006155 33840255

[53] Park AL, Ebesutani CK, Bose D, Chorpita BF. Psychometric Properties of a Spanish Translation of the Revised Child Anxiety and Depression Scale – Parent Version. J Psychopathol Behav Assess. 2015;38(2):307–19. doi: 10.1007/s10862-015-9517-7 PMC6197808 30364688

[54] Mishra P, Udas A, Sen S, Haldar K. Adapting a revised child anxiety and depression scale for rural india: a pilot, amenable to scale up. 2016. doi: 10.31234/io/25pnr

[55] Ryder AG, Yang J, Zhu X, Yao S, Yi J, Heine SJ, et al. The cultural shaping of depression: Somatic symptoms in China, psychological symptoms in North America?. Journal of Abnormal Psychology. 2008;117(2):300–13. doi: 10.1037/0021-843X.117.2.300 18489206

[56] Stodolska M, Livengood JS. The Influence of Religion on the Leisure Behavior of Immigrant Muslims in the United States. Journal of Leisure Research. 2006;38(3):293–320. doi: 10.1080/00222216.2006.11950080

[57] Datta S, Kingdon GG. Inequality in internet access in India: Implications for learning during COVID. 15387. IZA. 2022.

[58] Flavell JH, Green FL, Flavell ER, Harris PL, Astington JW. Young children’s knowledge about thinking. Monographs of the society for research in child development. 1995:i-113. 7877641

[59] Heath S, Charles V, Crow G, Wiles R. Informed consent, gatekeepers and go‐betweens: negotiating consent in child and youth‐orientated institutions. Br Educ Res J. 2007;33(3):403-17.

[60] Charters E. The Use of Think-aloud Methods in Qualitative Research An Introduction to Think-aloud Methods. Brock Education Journal. 2003;12(2). doi: 10.26522/brocked.v12i2.38

[61] Gilhooly KJ. Individual differences in thinking-aloud performance. Current Psychology. 1986;5(4):328–34. doi: 10.1007/bf02686600

