# Supplementary material for: Towards cultural validation of the revised children’s anxiety and depression scale in Karnataka, India: Think aloud interviews among 13–17 year olds
Source: PLoS One. 2026 Aug 19;21(8):e0352705. doi: 10.1371/journal.pone.0352705 (PMC13489475; doi:10.1371/journal.pone.0352705)
Supplement: Supplementary file 1 [file pone.0352705.s001.docx]

**Appendix A.** Examples of a selection of problems experienced by participants when responding to the RCADS items.

| **RCADS Subscale** | **RCADS item** | **Re-read or stumbled** | **Problems understanding or answering the Item** | **Misinterpretation of Item** | **Additional Notes** |
| --- | --- | --- | --- | --- | --- |
| MDD | 6. Nothing is much fun anymore | One participant seriously stumbled when reading the question (P. F). | One participant stated that they did not understand the question “*I can’t understand question 6*” (P. J). | One participant did not answer the given question “*Fun means in college. No more fun.”* (P.H). | None. |
|  | 15. I have problems with my appetite | Three participants seriously stumbled when reading the question (P. C, H, L- L stumbled over the pronunciation of appetite and attempted to pronounce several times). | One participant questioned the meaning of the question: ‘*what means appetite*?’ (P. H). | One participant did not answer the given question and discussed their daily routine (P. C). | Although not a significant pause, several participants struggled with pronunciation of the word ‘appetite’. |
| GAD | 13. I worry that something awful will happen to someone in my family | One participant seriously stumbled when reading the question (P. F). | One participant’s response appeared confused ‘*Never. Yes, that is persistent. Sometime*s.’ (P. H). | One participant confused the meaning of ‘family’: *’Never. I don’t bother about my family, I only bother about my parents, brothers and sisters*.’ (P. E) | None. |
| PD | 3. when I have a problem I get a funny feeling in my stomach | One participant seriously stumbled when reading the question (P. F). | One participant stated that they didn’t understand the question: *‘I can’t understand question 3, can I move to 4?’* (P. J). | Two participants did not answer the given question and discussed unrelated topics (P. E, H) | None. |
|  | 14. I suddenly feel as if I can’t breathe when there is no reason for this | One participant seriously stumbled when reading the question (P. F). | One participant stated that they couldn’t understand the question ‘*I couldn’t understand that question.’* But then answered the question (P. C).  One participant stated that they didn’t understand the question ‘*I can’t understand this question; can I move to the next?*’ (P. J). | One participant interpreted ‘can’t breathe’ as death and one as illness: ‘*when we feel that our parents or somebody has got any problem we feel like that we should not be alive in this world.*’ (P. E).  ‘*Yes, sometimes, in the (inaudible) area you cannot breathe there and I go to hospital and treat me*’ (P. H). | None. |
| SoP | 4. I worry when I think I have done poorly at something | One participant seriously stumbled while reading the question (P. F). | None. | Two participant did not answer the given question; one discussed poor internet connection (P. H) and one stated ‘I don’t do the poor things’ (P. E) | None. |
| OCD | 16. I have to keep checking that I have done things right (like the switch is off, or the door is locked) | Two participants seriously stumbled while reading the question (P. F, I). | None. | Four participants misinterpreted the question and discussed things that they would normally do in daily life (P. D, E, F, K). One participant discussed checking switches in relation to energy problems in India ‘*Electricity and power is very important for us so we have to save it. So, I will always be checking in my home’* (P. K). | One participant response did not make sense in English (P. C). |
|  | 31. I have to think of special thoughts (like numbers or words) to stop bad things from happening | One participant re-read the question before answering (P. K). | One participants questioned the item: ‘*Because of how special thoughts, like number or words stop bad things from happening?*’ (P. H). | Two participants misinterpreted the meaning of the question and discussed stopping bad thoughts in general (P. J, K). | None. |
|  | 44. I have to do some things in just the way to stop bad things from happening | One participant seriously stumbled while reading the question (P. F).  One participants reread the question before answering (P. D). | None. | Five participants misinterpreted the meaning of the question; many focused on societal changes/ actions to stop bad things happening in society (P. D, E, F, H, K). |  |
