# Supplementary material for: Towards cultural validation of the revised children’s anxiety and depression scale in Karnataka, India: Think aloud interviews among 13–17 year olds
Source: PLoS One. 2026 Aug 19;21(8):e0352705. doi: 10.1371/journal.pone.0352705 (PMC13489475; doi:10.1371/journal.pone.0352705)
Supplement: Supplementary file 2 [file pone.0352705.s003.docx]

| Reviewer | Comment | Response |
| --- | --- | --- |
| **Reviewer 1**  Responses to review 1 are highlighted in purple in the manuscript | Page 12 / Line 187: “answered on a four-point Likert scale depending on the frequency that the youth experiences [the symptom].” It may be more accurate to describe this as a four-point ordinal scale, as a Likert scale typically implies a neutral middle point. | ‘Likert’ has been deleted, so this now reads ‘answered on a four-point scale’ |
|  | Page 17 / Line 361: “Furthermore, participants were mostly from high SES backgrounds and living in urban areas.” Please define SES (socioeconomic status) for clarity. | The abbreviation of SES has been expanded on: Furthermore, participants were mostly from high Socio-Economic Status (SES) backgrounds and living in urban areas. |
|  | Throughout the manuscript: Obsessive Compulsive Disorder: Consider replacing “Obsessive Compulsive Disorder” with “OCD”, as the acronym is widely recognized even by non-expert audiences. | ‘Obsessive compulsive disorder’ has been replaced with OCD throughout the manuscript. |
| **Reviewer 2**  Blue  Responses already in the paper highlighted in green | **Major 1:**  Please clarify the study aim and terminology. The title says the word validation, and the term validation is referenced throughout, but it isn’t clear which procedures reflect establishing which type of validity. The study appears to focus on linguistic adaptation, and if this is the case, it would be helpful to revise the terminology throughout. For example, consider terms like “linguistic equivalence,” “semantic equivalence,” or “cultural relevance”. | **Response to the study title:**  While this study does not constitute a full psychometric validation of the Revised Children’s Anxiety and Depression Scale, the title uses the phrase ‘towards cultural validation’ intentionally to reflect an initial stage in the broader validation process. The think-aloud interviews specifically examined semantic and item equivalence, and generated evidence relating to response processes through participants’ interpretation and understanding of questionnaire items. Collectively, these aspects contribute to the cultural adaptation and validation literature. In addition, findings relating to OCD items provided preliminary insight into conceptual equivalence within the Karnataka context, further supporting the study’s contribution toward cultural validation rather than claiming completion of a full validation process.  **A section has been added to page 5 addressing what this study contributes to the validation process:**  However, it is acknowledged that this study does not constitute a full cultural validation or establish inferential equivalence. Rather, this study examines semantic equivalence, by assessing whether participants understood item wording as intended, and item equivalence, by exploring whether items were interpreted as relevant within participants’ lived contexts. The Think Aloud methodology also provided preliminary insight into participants’ response processes. These processes represent an important initial stage in the broader cultural adaptation and validation of the measure prior to psychometric testing.  **The language has been revised throughout e.g.:**  ‘aimed to establish the cultural and semantic equivalence’ – p.2  ‘and establishing the semantic equivalence’- p3  ‘aimed to examine the semantic, item, and response process equivalence of the RCADS-47 using cognitive interviewing methods with Indian adolescents, as a first stage towards its cultural validation’ - p. 10  **The aim has been clarified to reflect the change in language (p.5):**  The aim of this study was to examine the semantic, item, and response process equivalence of the RCADS-47 using cognitive interviewing methods with Indian adolescents, as a first stage towards its cultural validation. |
|  | **Major 2:**  please align the reader more pointedly to a process or framework that was followed, and how this study fits in a larger process. The authors state in the abstract this study is the first step in establishing cultural validity. However, a validation framework isn’t described. Authors refer the reader to steps 1 and 2 of the ITC (2017) Guidelines for Translating and Adapting tests. But, step 1 (permission for use of the tool in this study) is not described, and step 2 (consideration of linguistic, psychological, and cultural differences) is not clearly reported. If the ITC guidelines are the structure for the approach, consider to have the findings organized around these three aspects and present how the think-aloud data informed each. The authors report ascertaining item “comprehension” by participants. Notably, if that is the focus, this may better be described for readers as “linguistic” adaptation in the ITC terms. | We have now clarified the overarching validation framework guiding this study and more explicitly situated the present work within the broader process of cultural validation. Specifically, we revised the section to explain how this study aligns with Stages 1 and 2 of the ITC (2017) Guidelines and clarified that permission to use and adapt the measure was obtained from the copyright holders prior to commencing the study. Further to the ITC guidelines, Caron’s cultural validation guidelines were also used to develop the stages in the study, so it is consistent with a cultural validation study. This is outlined on page 4:  This study also drew upon the cultural validation guidelines presented by Caron (1999). This states that cultural validation is achieved through establishing the validity and reliability of the measure in the new population and consists of two stages. Broadly, cultural validations of mental health measures are reflective of this and are often conducted in two stages, with the first stage involving the verification of the cultural equivalence of the measure (Beaton et al., 2000; Caron, 1999; ICT, 2017), often in the form of a Think Aloud study, to determine ‘inferential equivalence’ within the target culture. Following this, psychometric testing is utilised to establish the measures validity and reliability in the target culture (Beaton et al., 2000; Caron, 1999: ICT, 2017). This study demonstrates Stage 1; verification of the cultural equivalence of the measure. This confirms that the measure maintains its ‘inferential equivalence’ within another culture, enabling it to accurately identify the constructs it was designed to.  A statement aligning with the ITC process stage 1 has been added to page 7:  Prior to commencement of the study, permission to use and culturally adapt the RCADS-47 was obtained from the copyright holders, consistent with Stage 1 of the ITC (2017) guidelines. |
|  | **Major 3:**  The manuscript would benefit from a clearer description of expert involvement. Step 2 of the ITC adaptation process requires the involvement of experts with relevant experience. Were local bilingual adolescent mental health professionals, clinical social workers, or counselors consulted, and what was their role during tool adaptation or interpretation of the data? | A section has been added to page 8 outlining the role of in country experts in the analysis:  Consistent with Step 2 of the ITC adaptation process, bilingual in-country mental health professionals from the SAMA project were involved throughout the adaptation process. Their expertise informed the interpretation of Think Aloud data, identification of potential semantic and cultural issues within items, and discussions regarding alternative phrasing to improve clarity and contextual relevance for adolescents in Karnataka. This expert input complemented the participant-based assessment of semantic and item equivalence presented in the current study. |
|  | **Major 4:**  Finally, a stated aim of this study is to establish “cultural equivalence” and this would include confirming construct validity, i.e. that the items are measuring the equivalent psychological construct, (the anxiety or depressive disorder symptom). It is unclear whether steps were taken to evaluate whether items measure equivalent psychological constructs. | A statement has been added to page 14 outlining the next stages and how cultural equivalence will be established:  A second stage of the validation process will involve psychometric evaluation of the revised measure within a larger sample of Indian adolescents. This will include examination of the reliability and construct validity of the adapted RCADS, including assessment of internal consistency, convergent and discriminant validity, and factor structure. The aim of this subsequent stage will be to determine whether the culturally adapted measure demonstrates appropriate psychometric properties and retains the intended anxiety and depression constructs within the Indian context |
|  | **Minor 1:**  Can the reference about the scale or burden of youth mental health problems in India be updated to one that is more recent than 2012? | More recent and robust statistics are limited, however, there is more recent data available regarding the youth suicide rate in India, which has been added to page 3:  India also has one of the highest youth suicide rates in the world (Patel et al., 2012; Senapati et al., 2024), with suicide being the 4th leading cause of adolescent death in India (Senapati et al., 2024). |
|  | **Minor 2:**  A first step in the references cited is translation. Was the tool “translated” from the original US English for English in Karnataka, or used “as-is” in US English? | The original US English version of the RCADS 47 was used in this study. This was originally made clear throughout the paper, along with the reasoning for using the original English version. Some examples of this are given below:  Page 2: to establish the cultural equivalence of the English-version of the RCADS-47  Page 5: Due to the diversity of languages spoken in India (121 legitimised languages; Jolad & Agarwal, 2021), we tested the English-version of the RCADS.  Page 5: Due to the large number of languages spoken in India (121 legitimised languages; Jolad & Agarwal, 2021), underlying religious connotation associated with different languages (e.g. Hindi is associated with Hinduism), and that in Karnataka young people are often taught in English in schools (with English also being a compulsory subject in all schools for Grade 5), validating the English version of the RCADS-47 was considered neutral and inclusive, alongside providing a good anchor language for further research. Per the 2011 census, English is the second most commonly spoken language in India, with the majority of these individuals speaking it as their second or third language (Ministry of Home Affairs India (2011). |
|  | **Minor 3:**  Line 156-157, there is a word missing, but I don’t follow the process of how “think-aloud” procedures “enabled” amendment of the wording (eg youth were asked to suggest alternate wording, or youth misunderstood and the research team then amended the wording). | A sentence has been added to page 8 summarising this process:  where difficulties in comprehension were identified, these terms were subsequently discussed with the Indian research team to identify more culturally and linguistically appropriate alternatives that would be more commonly understood within the target population. |
|  | **Minor 4:**  Were members of a youth advisory panel indicative of the “target population” in which this measure might be used, or is this a purposive or convenience sampling? How could it be a limitation and impact the data and findings? | A section has been added to page 6 addressing details about the youth advisory panel and sampling:  The Youth Advisory Group was purposively recruited for the SAMA project based on their interest in adolescent mental health and their enthusiasm to shape research on this in India. Group members did not have to have experienced poor mental health themselves. They were indicative of the target population in terms of age, being school-going, from a mix of urban and rural locales and speaking English as well as local languages. Recruitment via this YPAG for the TA study was convenience sampling.  A section discussing how this may be a limitation has been added to the strengths and limitations section (P.13):  A further limitation of the study may be that participants were recruited through the SAMA Youth Advisory Panel, whose members had a pre-existing interest in adolescent mental health and research involvement. Although the group was broadly reflective of the target population in terms of age, school attendance, geographic background, and language use, participants may have been more familiar with mental health concepts and terminology than adolescents in the wider population. As such, the study may underestimate potential difficulties in understanding certain items or the broader concept of mental health measurement among adolescents with less exposure to these topics. |
|  | **Minor 5:**  Participating youth had to be “experiencing positive mental wellbeing”. Is it a limitation, that this target population must not experience the mental health symptoms that the tool measures? | A section has been added to the methods section on page 6 detailing this inclusion decision:  This criterion was adopted in part due to safeguarding considerations, as the study was not designed or resourced to appropriately support adolescents experiencing significant or clinical levels of distress, in line with ethical guidance for research with vulnerable populations (Oates et al., 2021; World Health Organization, 2011). Although this may limit generalisability of findings, this study was intentionally focused on a non-clinical population. “*Positive emotional wellbeing*” was operationalised through adolescent self-identification at the point of recruitment. Participants and parents were provided, within the information sheet, with a brief and age-appropriate description of emotional wellbeing (e.g., generally feeling positive and not experiencing ongoing distress) and were advised to participate only if they felt this reflected their/ their child’s current experience. No formal screening tool was employed.  A discussion of this limitation has also been added to the discussion (p. 11):  Participants were recruited on the basis of self-identified positive emotional wellbeing, which may have introduced selection bias and restricts the generalisability of the findings. As a result, the perspectives captured are likely to reflect those of adolescents who are currently functioning well, and may not represent those experiencing emotional distress or clinical levels of difficulty. |
|  | **Minor 6:**  Please revise (or add) cultural adaptation references to reflect peer-reviewed publications on psychological tools. Consider key works on cultural equivalence, such as Flaherty et al. (1988), for example, which describes semantic, content, technical, criterion, and conceptual equivalence. (Flaherty, J. A., Gaviria, F. M., Pathak, D., Mitchell, T., Wintrob, R., Richman, J. A., & Birz, S. (1988). Developing instruments for cross-cultural psychiatric research. The Journal of nervous and mental disease, 176(5), 257–263.) | The work of Flaherty has been included and mentioned to support the procedure on pages 4-5:  This process is also informed by wider cross-cultural adaptation literature, including Flaherty et al. (1988), who identified several key dimensions of cultural equivalence in psychological tools, including semantic, content, technical, criterion, and conceptual equivalence.  In line with Flaherty et al. (1988), this represents an initial exploration of semantic and content equivalence within the target population. |
|  | **Minor 7:**  Since there is an approved tool in Hindi from the developers/ copyright owners, was the Hindi version consulted to inform adaptations for the cultural context? | An explanation regarding why the Hindi RCADS was not used to inform adaptions has been added to page 9:  The Hindi RCADS was not consulted as this study focused on semantic understanding of the English version among adolescents in Karnataka, which would have been obscured by relying on an already translated adaptation. Instead, in line with cultural adaptation guidelines, item interpretation was grounded directly in participants’ response processes (Beaton et al., 2000; International Test Commission, 2017). |
|  | **Minor 8:**  A limitation would seem to be the 30 minute interview duration, for young people ages 13 to 17 in Karnataka to learn the think aloud process and instructions, to read each item aloud, describe their answer and their reasons – and do this for each of 47 items. It would seem such a time limit for this process would not allow for rich data. | The decision for conducting interviews of this length has been addressed on page 7:  This was deemed an appropriate length of interview for this measure based on cognitive interviewing research and practice, as this study aimed to identify how adolescents understand and respond to items rather than obtaining lengthy narrative focusing on comprehension, interpretation, and reasoning (Ericsson & Simon, 1993). Research suggest that adequate responses can be obtained in this interview length (Ericsson & Simon, 1993). Shorter interviews have also been shown to reduce fatigue and help maintain adolescents’ engagement and concentration (Young & Schraedley, 2000). |
|  | **Minor 9:**  In results section, references that inform the methods or interpretations of findings may be better placed elsewhere. For example, lines 244-245 do not seem to belong in the findings, rather does this justify a method? | These sentences (As a general rule, we adopted a 25% cut-off, with items generating 3 or more problems (i.e. at least three participants having trouble with that item across any of the six coding categories) being candidates for rephrasing, similar to previous Think Aloud studies (e.g. Zhang et al., 2022).) have been moved to the methods section p7-8. |
|  | **Minor 10:**  Descriptions in the findings are using terms like “most” and “many” and heavily focused on numbers (of coded data). Can they be better framed as qualitative findings of a small qualitative study? | We agree that the presentation of findings differs somewhat from more traditional qualitative research approaches. However, Think Aloud studies conducted for measure adaptation and validation have a distinct methodological purpose, namely the identification and characterisation of comprehension, semantic, and response issues within questionnaire items. As such, reporting the number and types of identified issues is standard practice within cognitive interviewing and Think Aloud methodology, as this helps indicate the prevalence and potential significance of item-level problems within the sample. A sentence has been added to the method clarifying this (p.8):  Consistent with recommendations for cognitive interviewing and Think Aloud methodology in questionnaire adaptation research, the frequency and type of identified comprehension and interpretation issues were reported to indicate the relative prominence of item-level difficulties within the sample, rather than to imply statistical generalisability (Beatty & Willis, 2007; Willis & Miller, 2011; Meadows, 2021). |
|  | **Minor 11:**  The focus of the discussion is nearly exclusively on the OCD subscale. At the time of the RCADS development, OCD was classified as an anxiety disorder in the DSM. It no longer is, as the authors point out. Can the lack of current relevance of OCD symptoms as symptoms of anxiety be more clear, and the discussion highlight other important findings? | Additional discussion has been provided for both the MDD and PD subscales:  This problem was particularly evident within the MDD subscale. This difficulty may reflect cultural and linguistic differences in how depressive symptoms are conceptualised and expressed among Indian adolescents. Psychological terminology, as well as commonly used Western terms, may not translate directly across cultures, particularly for items describing internal or somatic experiences (Flaherty et al., 1988; Beaton et al., 2000). For example, terms such as “appetite” (Item 15) may be less commonly used or interpreted differently within everyday adolescent language in the Indian context. This is particularly relevant for the MDD subscale, where depressive symptoms often include somatic and internally experienced states that may be described differently across cultures, potentially affecting how items are understood and endorsed. P. 12  This extends to the PD subscale, which may reflect cultural differences in how acute anxiety is experienced and labelled, with symptoms often expressed in somatic or contextual terms rather than as “panic” in a clinical sense, such as dizziness, breathlessness etc. (Kleinman, 1987; Ryder et al., 2008). This, alongside limited familiarity with diagnostic terminology, may lead to misunderstanding of item wording during. P. 12  Alongside, acknowledgement of the reclassification of OCD:  Although the OCD subscale was a prominent focus within the findings, OCD is no longer classified as an anxiety disorder within the DSM-5, despite its inclusion in the original RCADS framework at the time of development. Therefore, the OCD findings should be interpreted cautiously. P. 11 |
| **Reviewer 3**  Responses to reviewer 3 are indicated in Yellow | **Introduction:** 1. The rationale for cultural validation is well-articulated. However, the introduction could benefit from a clearer statement of how this study builds on previous validation efforts in India (e.g., Long et al., 2013).  2. Some references have future dates (e.g., Ali et al., 2026). Please verify and correct. | A statement of how this build on previous research ahs been added to page 4:  Previously Long et al. (2013) psychometrically tested the unmodified RCADS with a sample of Indian adolescents, to establish factor structure, this study will build on this examining Indian adolescents’ interpretations of items to identify linguistic and cultural discrepancies, thereby ensuring the measure more accurately captures culturally grounded experiences of anxiety and depression.  Future references (due to typos) have been amended:  Ali, G. C., Ryan, G., & De Silva, M. J. Validated screening tools for common mental disorders in low and middle income countries: a systematic review. *PloS one*. 2016, *11*(6), e0156939. |
|  | **Methods:**  1.**Sample:** The sample size (n=12) is acceptable for a Think Aloud study, but the demographic skew toward high SES and urban adolescents limits generalizability. This limitation is acknowledged but should be emphasized more strongly in the discussion.  2. **Age Range:** The target age was 13–17 years, yet no 13-year-olds were included. Please clarify why and discuss implications.  3. **Coding:** The coding framework is sound, and inter-rater reliability (K=0.75) is acceptable. However, the threshold for identifying problematic items (≥3 participants) needs stronger justification—why was 25% chosen? | **1.Sample:** The section discussing participants SES in the discussion section has been expanded on further in the discussion (p. 12) to emphasis this limitation:  This homogeneity may be partly due to the study being conducted online during COVID-19 travel restrictions, resulting in a sample consisting solely of adolescents with internet access. Consequently, adolescents from lower SES backgrounds were underrepresented, substantially limiting the generalizability of findings to the broader adolescent population in Karnataka and India more widely. This limitation is particularly important given the well-documented digital divide in India, where internet access differs significantly according to socioeconomic status and rural–urban location (Datta & Kingdon, 2022). Adolescents from urban, high-SES backgrounds may also have had greater exposure to English and higher educational opportunities, potentially reducing the identification of language and comprehension difficulties within the RCADS items. Previous research has shown that digital exclusion and educational inequalities may disproportionately affect rural adolescents and those from disadvantaged backgrounds (Datta & Kingdon, 2022; Venkareddy, 2024). Therefore, future validation studies should prioritize recruiting adolescents from rural and lower-SES populations to improve representativeness and cultural applicability of the RCADS-47. A further limitation is that, although this study is a step towards validating the RCADS-47 in adolescents aged 13–17 years, no 13-year-olds were recruited at this stage of the study.  **2.Age Range**: A section has been added in explaining this on page 14:  The absence of 13-year-old participants is a limitation as it reduces the representativeness of the sample across the full 13–17 age range and limits generalisability of findings to younger adolescents. No 13-year-olds were included in this study as none opted in to take part, despite the study being advertised to this age group. This is important because younger adolescents may differ in cognitive and linguistic development, which can affect how they interpret and articulate responses during Think Aloud tasks (Flavell, 1992; Erikson, 1968). Their non-participation may reflect lower consent uptake or engagement among younger adolescents, a common issue in adolescent mental health research (Heath et al., 2004).  **3. Coding:** A section has been added to page 8 with further justification for the 25% cut off:  This threshold was selected to balance sensitivity and specificity, ensuring that item revision was driven by systematic patterns of difficulty rather than isolated or idiosyncratic misunderstandings. Within Cognitive Interviewing, there is no universally prescribed numerical criterion; however, methodological guidance emphasises the importance of identifying recurrent response problems across participants as evidence of substantive threats to Response Process Validity (e.g., Willis, 2005; Beatty & Willis, 2007). Given typical sample sizes in think-aloud studies (often 10–15 participants), a threshold of three or more participants has been recommended as indicating meaningful and non-trivial comprehension or interpretation issues (e.g., Blair & Conrad, 2011). Thus, the 25% criterion provides a pragmatic and empirically grounded approach to identifying problematic items while avoiding over-modification based on limited evidence. |
|  | **Results:**  **Tables:** Tables are informative, but Table 4 could include a column explaining the rationale for each rephrasing. | A brief rationale has been added to Table 4 for each item p. 38-40. |
|  | **Discussion:**  **1. OCD explanations:** The explanation of OCD-related issues would benefit from more empirical references on cultural symptom presentation.  **2. English language:** The impact of Indian English dialect and language proficiency is noted but could be expanded with examples from the data.  **3. Stage 2 Plan:** The authors should outline a clear plan for Stage 2 (psychometric testing) in the conclusion.  **4. Discussion points expansion:** Expanded discussion on sample bias and cultural implications. | **1.OCD explanations:** The paragraph regarding OCD has been rewritten to include empirical referenced to cultural symptom presentation (p. 9-10):  First, research indicates that symptoms of Obsessive-Compulsive Disorder can vary cross-culturally, with differences in symptom content, expression, and severity reported between non-Western and Western populations (e.g., Nicolini & Salin-Pascual, 2017; Sharma et al., 2019). Studies conducted in Indian samples have identified distinct clinical presentations, including greater prominence of religious and contamination-related obsessions, as well as culturally embedded compulsive behaviours (Sharma et al., 2019). As the RCADS was developed in the United States based on Western conceptualisations of OCD, some item content may not adequately reflect culturally specific manifestations of symptoms in Indian adolescents. For example, repetitive cleaning behaviours (e.g., Item 42) are commonly interpreted as pathological within Western diagnostic frameworks; however, in the Indian context, practices related to cleanliness are often embedded within religious and cultural norms, particularly within Hinduism (Dhand, 2002). Empirical research further suggests that higher levels of religiosity are associated with increased endorsement of OCD-like traits (Agorastos et al., 2014), raising the possibility that such items may conflate culturally normative behaviours with psychopathology in certain populations. While the relationship between religiosity and OCD is complex and multifaceted (Agorastos et al., 2014), these findings highlight potential challenges in the cross-cultural validity of OCD subscales.  **2. English language:** The sentence ‘Culture influences the teaching and speaking of English (Wang, 2011), generating dialects, of which Indian English is one.’ (p.12) has been removed to avoid confusion around the use of ‘dialect’ and additional examples of language confusion have been added to page 12.  **3. Stage 2 plan:** A summary of stage 2 has been added to the discussion section (p. 14):  A second stage of the validation process will involve psychometric evaluation of the revised measure within a larger sample of Indian adolescents. This will include examination of the reliability and construct validity of the adapted RCADS, including assessment of internal consistency, convergent and discriminant validity, and factor structure. The aim of this subsequent stage will be to determine whether the culturally adapted measure demonstrates appropriate psychometric properties and retains the intended anxiety and depression constructs within the Indian context  **4. Discussion points expansion:**  Limitations have been expanded regarding sample bias and cultural implications:  **Sample Bias:**  Participants were recruited on the basis of self-identified positive emotional wellbeing, which may have introduced selection bias and restricts the generalisability of the findings. As a result, the perspectives captured are likely to reflect those of adolescents who are currently functioning well, and may not represent those experiencing emotional distress or clinical levels of difficulty. (p. 12)  A further limitation of the study may be that participants were recruited through the SAMA Youth Advisory Panel, whose members had a pre-existing interest in adolescent mental health and research involvement. Although the group was broadly reflective of the target population in terms of age, school attendance, geographic background, and language use, participants may have been more familiar with mental health concepts and terminology than adolescents in the wider population. As such, the study may underestimate potential difficulties in understanding certain items or the broader concept of mental health measurement among adolescents with less exposure to these topics. (p. 13)  This homogeneity may be partly due to the study being conducted online during COVID-19 travel restrictions, resulting in a sample consisting solely of adolescents with internet access. Consequently, adolescents from lower SES backgrounds and rural areas were underrepresented, substantially limiting the generalizability of findings to the broader adolescent population in Karnataka and India more widely. This limitation is particularly important given the well-documented digital divide in India, where internet access differs significantly according to socioeconomic status and rural–urban location (Datta & Kingdon, 2022). Adolescents from urban, high-SES backgrounds may also have had greater exposure to English and higher educational opportunities, potentially reducing the identification of language and comprehension difficulties within the RCADS items. Previous research has shown that digital exclusion and educational inequalities may disproportionately affect rural adolescents and those from disadvantaged backgrounds (Datta & Kingdon, 2022; Venkareddy, 2024). Therefore, future validation studies should prioritize recruiting adolescents from rural and lower-SES populations to improve representativeness and cultural applicability of the RCADS-47. A further limitation is that, although this study is a step towards validating the RCADS-47 in adolescents aged 13–17 years, no 13-year-olds were recruited at this stage of the study. (P. 12)  **Cultural implications:**  More broadly, India is highly culturally, linguistically, and socioeconomically diverse, and understandings, expressions, and communication of mental health symptoms may vary substantially across regions, communities, family structures, and social groups (e.g. Gaiha et al., 2020). Cultural norms relating to emotional expression, family relationships, academic expectations, social responsibility, and stigma surrounding mental health may all influence how adolescents interpret questionnaire items and decide upon responses. Consequently, certain RCADS items may hold different meanings, levels of relevance, or emotional salience in different Indian contexts. Although the current study provides preliminary evidence of semantic and item equivalence among adolescents in Karnataka, further work is needed to explore whether similar interpretations are observed across adolescents from different linguistic, religious, rural/urban, and socioeconomic backgrounds within India. (p. 12) |
|  | **References:** Ensure all URLs and DOIs are accessible. | These have been reviewed and updated in the reference section. |
| **Reviewer 4** | The authors did identify several problems regarding the RCADS-47, especially for the Obsessive-Compulsive Disorder Subscale. Since the authors have already identified the problems, I suggest this paper should outline the modifications of the items made to address these problems, laying the groundwork for the subsequent step “confirmation.” | These adaptions have been outlined in Table 4 (Page 37), mentioned on Pag 9 of the discussion.  Suggested rephrasing is shown in Table 4 |
