# Supplementary material for: Towards cultural validation of the revised children’s anxiety and depression scale in Karnataka, India: Think aloud interviews among 13–17 year olds
Source: PLoS One. 2026 Aug 19;21(8):e0352705. doi: 10.1371/journal.pone.0352705 (PMC13489475; doi:10.1371/journal.pone.0352705)
Supplement: Peer review history

# Peer review: Decision Letter 0

**DOI:** 10.1371/journal.pone.0352705.r001

**Authors:** Mu-Hong Chen

1 Dec 2025

Dear Dr. Palmer,

Thank you for submitting your manuscript to PLOS ONE. After careful consideration, we feel that it has merit but does not fully meet PLOS ONE’s publication criteria as it currently stands. Therefore, we invite you to submit a revised version of the manuscript that addresses the points raised during the review process.

Please submit your revised manuscript by Jan 15 2026 11:59PM. If you will need more time than this to complete your revisions, please reply to this message or contact the journal office at plosone@plos.org. When you're ready to submit your revision, log on to https://www.editorialmanager.com/pone/ and select the 'Submissions Needing Revision' folder to locate your manuscript file.

- A rebuttal letter that responds to each point raised by the academic editor and reviewer(s). You should upload this letter as a separate file labeled 'Response to Reviewers'.
- A marked-up copy of your manuscript that highlights changes made to the original version. You should upload this as a separate file labeled 'Revised Manuscript with Track Changes'.
- An unmarked version of your revised paper without tracked changes. You should upload this as a separate file labeled 'Manuscript'.

If you would like to make changes to your financial disclosure, please include your updated statement in your cover letter. Guidelines for resubmitting your figure files are available below the reviewer comments at the end of this letter.

If applicable, we recommend that you deposit your laboratory protocols in protocols.io to enhance the reproducibility of your results. Protocols.io assigns your protocol its own identifier (DOI) so that it can be cited independently in the future. For instructions see: https://journals.plos.org/plosone/s/submission-guidelines#loc-laboratory-protocols. Additionally, PLOS ONE offers an option for publishing peer-reviewed Lab Protocol articles, which describe protocols hosted on protocols.io. Read more information on sharing protocols at https://plos.org/protocols?utm_medium=editorial-email&utm_source=authorletters&utm_campaign=protocols.

We look forward to receiving your revised manuscript.

Kind regards,

Mu-Hong Chen, M.D., Ph.D.

Academic Editor

PLOS ONE

**Journal Requirements:**

When submitting your revision, we need you to address these additional requirements.

1. Please ensure that your manuscript meets PLOS ONE's style requirements, including those for file naming. The PLOS ONE style templates can be found at

https://journals.plos.org/plosone/s/file?id=wjVg/PLOSOne_formatting_sample_main_body.pdf and

https://journals.plos.org/plosone/s/file?id=ba62/PLOSOne_formatting_sample_title_authors_affiliations.pdf

2. In the online submission form you indicate that your data is not available for proprietary reasons and have provided a contact point for accessing this data. Please note that your current contact point is a co-author on this manuscript. According to our Data Policy, the contact point must not be an author on the manuscript and must be an institutional contact, ideally not an individual. Please revise your data statement to a non-author institutional point of contact, such as a data access or ethics committee, and send this to us via return email. Please also include contact information for the third party organization, and please include the full citation of where the data can be found.

3. If the reviewer comments include a recommendation to cite specific previously published works, please review and evaluate these publications to determine whether they are relevant and should be cited. There is no requirement to cite these works unless the editor has indicated otherwise.

[Note: HTML markup is below. Please do not edit.]

Reviewers' comments:

Reviewer's Responses to Questions

**Comments to the Author**

1. Is the manuscript technically sound, and do the data support the conclusions?

Reviewer #1: Yes

Reviewer #2: Partly

Reviewer #3: Yes

Reviewer #4: Yes

**********

2. Has the statistical analysis been performed appropriately and rigorously?

Reviewer #1: N/A

Reviewer #2: N/A

Reviewer #3: Yes

Reviewer #4: N/A

**********

3. Have the authors made all data underlying the findings in their manuscript fully available?

The PLOS Data policy

Reviewer #1: Yes

Reviewer #2: No

Reviewer #3: Yes

Reviewer #4: Yes

**********

4. Is the manuscript presented in an intelligible fashion and written in standard English?

Reviewer #1: Yes

Reviewer #2: Yes

Reviewer #3: Yes

Reviewer #4: Yes

**********

Reviewer #1: In my opinion, this is a methodologically sound piece of work, and the report is excellent. The authors clearly acknowledge the limitations, particularly the skewed representation of Indian adolescents in relation to language and education, yet I find the findings important even within these constraints. The procedure described is rigorous, albeit demanding. I do wonder, however, whether the lack of understanding of certain items might be attributed to the fact that the participants themselves did not experience the disorders. It would be valuable if the authors could comment on this point. Although the tool is designed as a screening measure, individuals who experience the traits (either subclinically or clinically) would likely have had a better understanding of the symptoms described in the scale.

Please find below some minor comments:

Page 12 / Line 187: “answered on a four-point Likert scale depending on the frequency that the youth experiences [the symptom].” It may be more accurate to describe this as a four-point ordinal scale, as a Likert scale typically implies a neutral middle point.

Page 17 / Line 361: “Furthermore, participants were mostly from high SES backgrounds and living in urban areas.” Please define SES (socioeconomic status) for clarity.

Throughout the manuscript: Obsessive Compulsive Disorder: Consider replacing “Obsessive Compulsive Disorder” with “OCD”, as the acronym is widely recognized even by non-expert audiences.

Reviewer #2: Thank you for the opportunity to review this manuscript. This paper describes think-aloud interviews conducted in Karnataka, India of the Revised Children’s Anxiety and Depression Scale (RCADS) in the English language. It is important work, and evidence for culturally adapted and validated tools is needed. Overall, the writing is generally clear and concise. Methodologically, my main concern is the procedures described do not align with the “steps” or process cited. It is also not clear in the manuscript as to what aspect(s) of translation, adaptation or validation was completed using this assessment of think-aloud interview data.

MAJOR:

Please clarify the study aim and terminology. The title says the word validation, and the term validation is referenced throughout, but it isn’t clear which procedures reflect establishing which type of validity. The study appears to focus on linguistic adaptation, and if this is the case, it would be helpful to revise the terminology throughout. For example, consider terms like “linguistic equivalence,” “semantic equivalence,” or “cultural relevance”.

Second, please align the reader more pointedly to a process or framework that was followed, and how this study fits in a larger process. The authors state in the abstract this study is the first step in establishing cultural validity. However, a validation framework isn’t described. Authors refer the reader to steps 1 and 2 of the ITC (2017) Guidelines for Translating and Adapting tests. But, step 1 (permission for use of the tool in this study) is not described, and step 2 (consideration of linguistic, psychological, and cultural differences) is not clearly reported. If the ITC guidelines are the structure for the approach, consider to have the findings organized around these three aspects and present how the think-aloud data informed each. The authors report ascertaining item “comprehension” by participants. Notably, if that is the focus, this may better be described for readers as “linguistic” adaptation in the ITC terms.

Third, the manuscript would benefit from a clearer description of expert involvement. Step 2 of the ITC adaptation process requires the involvement of experts with relevant experience. Were local bilingual adolescent mental health professionals, clinical social workers, or counselors consulted, and what was their role during tool adaptation or interpretation of the data?

Finally, a stated aim of this study is to establish “cultural equivalence” and this would include confirming construct validity, i.e. that the items are measuring the equivalent psychological construct, (the anxiety or depressive disorder symptom). It is unclear whether steps were taken to evaluate whether items measure equivalent psychological constructs.

In short, ascertaining “comprehension” of items via think-aloud methods seems to be well described. It may be clearer for readers if the terminology used and aims center on this linguistic focus.

MINOR:

--Can the reference about the scale or burden of youth mental health problems in India be updated to one that is more recent than 2012?

--A first step in the references cited is translation. Was the tool “translated” from the original US English for English in Karnataka, or used “as-is” in US English?

--Line 156-157, there is a word missing, but I don’t follow the process of how “think-aloud” procedures “enabled” amendment of the wording (eg youth were asked to suggest alternate wording, or youth misunderstood and the research team then amended the wording).

--Were members of a youth advisory panel indicative of the “target population” in which this measure might be used, or is this a purposive or convenience sampling? How could it be a limitation and impact the data and findings?

--Participating youth had to be “experiencing positive mental wellbeing”. Is it a limitation, that this target population must not experience the mental health symptoms that the tool measures?

--Please revise (or add) cultural adaptation references to reflect peer-reviewed publications on psychological tools. Consider key works on cultural equivalence, such as Flaherty et al. (1988), for example, which describes semantic, content, technical, criterion, and conceptual equivalence. (Flaherty, J. A., Gaviria, F. M., Pathak, D., Mitchell, T., Wintrob, R., Richman, J. A., & Birz, S. (1988). Developing instruments for cross-cultural psychiatric research. The Journal of nervous and mental disease, 176(5), 257–263.)

--Since there is an approved tool in Hindi from the developers/ copyright owners, was the Hindi version consulted to inform adaptations for the cultural context?

--A limitation would seem to be the 30 minute interview duration, for young people ages 13 to 17 in Karnataka to learn the think aloud process and instructions, to read each item aloud, describe their answer and their reasons – and do this for each of 47 items. It would seem such a time limit for this process would not allow for rich data.

--In results section, references that inform the methods or interpretations of findings may be better placed elsewhere. For example, lines 244-245 do not seem to belong in the findings, rather does this justify a method?

--Descriptions in the findings are using terms like “most” and “many” and heavily focused on numbers (of coded data). Can they be better framed as qualitative findings of a small qualitative study?

--The focus of the discussion is nearly exclusively on the OCD subscale. At the time of the RCADS development, OCD was classified as an anxiety disorder in the DSM. It no longer is, as the authors point out. Can the lack of current relevance of OCD symptoms as symptoms of anxiety be more clear, and the discussion highlight other important findings?

Reviewer #3: General Comments

This manuscript addresses an important gap in adolescent mental health research in India by initiating cultural validation of the RCADS-47. The study is timely and relevant, given the high prevalence of anxiety and depression among young people and the lack of culturally validated tools. The use of Think Aloud interviews is appropriate for assessing item comprehension and cultural equivalence.

Introduction

• The rationale for cultural validation is well-articulated. However, the introduction could benefit from a clearer statement of how this study builds on previous validation efforts in India (e.g., Long et al., 2013).

• Some references have future dates (e.g., Ali et al., 2026). Please verify and correct.

Methods

• Sample: The sample size (n=12) is acceptable for a Think Aloud study, but the demographic skew toward high SES and urban adolescents limits generalizability. This limitation is acknowledged but should be emphasized more strongly in the discussion.

• Age Range: The target age was 13–17 years, yet no 13-year-olds were included. Please clarify why and discuss implications.

• Coding: The coding framework is sound, and inter-rater reliability (K=0.75) is acceptable. However, the threshold for identifying problematic items (≥3 participants) needs stronger justification—why was 25% chosen?

Results

• The presentation of problematic items and suggested rephrasing is clear and useful. Consider summarizing key examples from Appendix A in the main text for better readability.

• Tables are informative, but Table 4 could include a column explaining the rationale for each rephrasing.

Discussion

• The discussion insightfully links cultural norms (e.g., religiosity, COVID-19 behavioral changes) to item misinterpretation. However:

o The explanation of OCD-related issues would benefit from more empirical references on cultural symptom presentation.

o The impact of Indian English dialect and language proficiency is noted but could be expanded with examples from the data.

• The authors should outline a clear plan for Stage 2 (psychometric testing) in the conclusion.

Ethics

• Ethical safeguards and approvals are well-documented. The inclusion of wellbeing checks and consent procedures is commendable.

References

• Several references have incorrect or future publication dates—please verify.

• Ensure all URLs and DOIs are accessible.

Strengths

• Addresses a critical gap in adolescent mental health research in India.

• Transparent methodology and ethical rigor.

• Provides actionable recommendations for item rephrasing.

Limitations

• Small, homogeneous sample limits generalizability.

• Online recruitment may bias toward higher SES and English proficiency.

• Lack of psychometric data at this stage (acknowledged as Stage 1).

Recommendation

Major Revision

The manuscript is promising and relevant but requires:

• Stronger justification for methodological choices (e.g., item threshold).

• Expanded discussion on sample bias and cultural implications.

• Correction of reference inconsistencies.

• Clearer roadmap for Stage 2 validation.

Reviewer #4: This study aimed to establish the cultural equivalence of the English-version of the Revised Children’s Anxiety and Depression Scale (RCADS-47) in a sample of Indian adolescents. This study focuses on stages 1 (pre-condition) and 2 (test development) of the guidelines proposed by the International Test Commission.

The authors did identify several problems regarding the RCADS-47, especially for the Obsessive-Compulsive Disorder Subscale. Since the authors have already identified the problems, I suggest this paper should outline the modifications of the items made to address these problems, laying the groundwork for the subsequent step “confirmation.”

**********

what does this mean?). If published, this will include your full peer review and any attached files.

If you choose “no”, your identity will remain anonymous but your review may still be made public.

**Do you want your identity to be public for this peer review?**  For information about this choice, including consent withdrawal, please see our Privacy Policy

Reviewer #1: No

Reviewer #2: No

Reviewer #3: **Yes:** G. Nduku Wambua

Reviewer #4: No

**********

[NOTE: If reviewer comments were submitted as an attachment file, they will be attached to this email and accessible via the submission site. Please log into your account, locate the manuscript record, and check for the action link "View Attachments". If this link does not appear, there are no attachment files.]

To ensure your figures meet our technical requirements, please review our figure guidelines: https://journals.plos.org/plosone/s/figures

You may also use PLOS’s free figure tool, NAAS, to help you prepare publication quality figures: https://journals.plos.org/plosone/s/figures#loc-tools-for-figure-preparation.

NAAS will assess whether your figures meet our technical requirements by comparing each figure against our figure specifications.

# Author comment: Author response to Decision Letter 1

**DOI:** 10.1371/journal.pone.0352705.r002

2 Jun 2026

Comments are responded to in the attached document.

Attachment Submitted filename: TA PO reviewer comments.docx

# Peer review: Decision Letter 1

**DOI:** 10.1371/journal.pone.0352705.r003

**Authors:** Mu-Hong Chen

14 Jun 2026

Towards Cultural Validation of the Revised Children’s Anxiety and Depression Scale in Karnataka, India: Think Aloud Interviews Among 13-17 Year Olds

PONE-D-25-30808R1

Dear Dr. Amy Palmer,

We’re pleased to inform you that your manuscript has been judged scientifically suitable for publication and will be formally accepted for publication once it meets all outstanding technical requirements.

Within one week, you’ll receive an e-mail detailing the required amendments. When these have been addressed, you’ll receive a formal acceptance letter and your manuscript will be scheduled for publication.

An invoice will be generated when your article is formally accepted. Please note, if your institution has a publishing partnership with PLOS and your article meets the relevant criteria, all or part of your publication costs will be covered. Please make sure your user information is up-to-date by logging into Editorial Manager at Editorial Manager® and clicking the ‘Update My Information' link at the top of the page. For questions related to billing, please contact billing support.

If your institution or institutions have a press office, please notify them about your upcoming paper to help maximize its impact. If they’ll be preparing press materials, please inform our press team as soon as possible -- no later than 48 hours after receiving the formal acceptance. Your manuscript will remain under strict press embargo until 2 pm Eastern Time on the date of publication. For more information, please contact onepress@plos.org.

Kind regards,

Mu-Hong Chen, M.D., Ph.D.

Academic Editor

PLOS One

Additional Editor Comments (optional):

Reviewers' comments:

Reviewer's Responses to Questions

**Comments to the Author**

Reviewer #1: All comments have been addressed

Reviewer #2: All comments have been addressed

Reviewer #4: (No Response)

**********

2. Is the manuscript technically sound, and do the data support the conclusions?

Reviewer #1: Yes

Reviewer #2: Yes

Reviewer #4: (No Response)

**********

3. Has the statistical analysis been performed appropriately and rigorously?

Reviewer #1: Yes

Reviewer #2: N/A

Reviewer #4: (No Response)

**********

4. Have the authors made all data underlying the findings in their manuscript fully available?

The PLOS Data policy

Reviewer #1: Yes

Reviewer #2: (No Response)

Reviewer #4: (No Response)

**********

5. Is the manuscript presented in an intelligible fashion and written in standard English?

Reviewer #1: Yes

Reviewer #2: Yes

Reviewer #4: (No Response)

**********

Reviewer #1: All comments were successfully addressed, for all three reviewers in my opinion.

Reviewer #2: I commend the authors for a very thorough job in responding to and satisfying all of my comments. It is a very strong paper now and will be a great contribution to the literature.

Reviewer #4: (No Response)

**********

what does this mean?). If published, this will include your full peer review and any attached files.

If you choose “no”, your identity will remain anonymous but your review may still be made public.

**Do you want your identity to be public for this peer review?**  For information about this choice, including consent withdrawal, please see our Privacy Policy

Reviewer #1: No

Reviewer #2: No

Reviewer #4: No

**********

# Editor report: Acceptance letter

**DOI:** 10.1371/journal.pone.0352705.r004

**Authors:** Mu-Hong Chen

PONE-D-25-30808R1

PLOS One

Dear Dr. Palmer,

I'm pleased to inform you that your manuscript has been deemed suitable for publication in PLOS One. Congratulations! Your manuscript is now being handed over to our production team.

At this stage, our production department will prepare your paper for publication. This includes ensuring the following:

* All references, tables, and figures are properly cited

* All relevant supporting information is included in the manuscript submission,

* There are no issues that prevent the paper from being properly typeset

You will receive further instructions from the production team, including instructions on how to review your proof when it is ready. Please keep in mind that we are working through a large volume of accepted articles, so please give us a few days to review your paper and let you know the next and final steps.

Lastly, if your institution or institutions have a press office, please let them know about your upcoming paper now to help maximize its impact. If they'll be preparing press materials, please inform our press team within the next 48 hours. Your manuscript will remain under strict press embargo until 2 pm Eastern Time on the date of publication. For more information, please contact onepress@plos.org.

You will receive an invoice from PLOS for your publication fee after your manuscript has reached the completed accept phase. If you receive an email requesting payment before acceptance or for any other service, this may be a phishing scheme. Learn how to identify phishing emails and protect your accounts at https://explore.plos.org/phishing.

If we can help with anything else, please email us at customercare@plos.org.

Thank you for submitting your work to PLOS ONE and supporting open access.

Kind regards,

PLOS ONE Editorial Office Staff

on behalf of

Dr. Mu-Hong Chen

Academic Editor

PLOS One
